# Nondairy ice cream based on fermented yam (*Dioscorea* sp.)

**DOI:** 10.1002/fsn3.1051

**Published:** 2019-04-23

**Authors:** Nádia N. Batista, Cíntia L. Ramos, Josiane F. Pires, Silvino I. Moreira, Eduardo Alves, Disney R. Dias, Rosane F. Schwan

**Affiliations:** ^1^ Department of Food Science Federal University of Lavras Lavras Brazil; ^2^ Department of Biology Federal University of Lavras Lavras Brazil; ^3^ Department of Basic Science Federal University of Vales do Jequitinhonha e Mucuri Diamantina Brazil; ^4^ Department of Plant Pathology Federal University of Lavras Lavras Brazil

**Keywords:** yam, lactic acid bacteria, minerals, TPC, antioxidant activity, ice cream

## Abstract

**Background:**

The demand for industrialized foods that contribute to health and well‐being has characterized the new generation of consumers. Yam (*Dioscorea* sp.) is a nutritious food; however, it is not used very much in industrial food processes. The objective of this study was to develop and to characterize a truly dairy‐free low‐fat ice cream prepared from unfermented and fermented with yam dough.

**Results:**

The fermentation was conducted by *Leuconostoc lactic* CCMA 0415 remained viable (10^7^ CFU/g) during 90 days of storage. The fermentation process reduced the starch concentration from 26.82% to 22.35% and the protein concentration from 4.68% to 3.99% and increased the concentration of some minerals (K, S, Cu, Mn, Zn, and Fe). The total phenolic contents for fermented and unfermented ice creams were 51 and 54 mg, respectively. The radical scavenging activity were 18% and 10% with the 1,1‐diphenyl‐2‐picrylhydrazyl method and 44% and 26% with the 2,2’‐azino‐bis (3 ethylbenzo‐thiazoline‐6‐sulfonic acid) method for the unfermented and fermented samples, respectively. The fermented and unfermented ice creams were both characterized as non‐Newtonian fluids exhibiting pseudoplastic behaviors.

**Conclusions:**

These results indicated that yam is a suitable substitute for milk, thus making it an option to produce edible lactose‐free ice cream with low fat.

## 1 INTRODUCTION

Yam (*Dioscorea* sp.) is a nutritious food; however, it is not used very much in industrial food processes. It has been commercialized mainly as an *in natura* product. Approximately 62 million tons of yam is annually produced, and around 6.7 million tons are lost during postharvest treatment (FAO, 2013). Yam processing is an alternative to reduce waste and increase the consumption of this tuber by the population.

Antinutritional compounds, such as phytate and oxalate, derivates of phytic and oxalic acids, respectively, are commonly found in plants, including yam plants. These compounds bind to minerals and proteins, thus decreasing their availability in food. However, fermentation and cooking have been shown to reduce the concentrations of antinutritional compounds in plant‐based foods (Patel, Gadewar, Tahilyani, & Patel, 2012; Peng et al., 2011).

Among the microbial groups that can reduce the concentrations of these antinutritional compounds by fermentation, the lactic acid bacteria (LAB) are highlighted for their metabolic versatility. In addition to produce enzymes, this bacterial group can ferment a variety of substrates and produce organic acids, bacteriocins, and exopolysaccharides. In addition, some strains exhibit probiotic properties (Liu, Han, & Zhou, 2011).

The genus *Leuconostoc* (*Leuc*.) is “generally recognized as safe” (GRAS), and many species are efficient producers of mannitol and class II bacteriocin (Eijsink et al., 2002; Ortiz, Bleckwedel, Raya, & Mozzi, 2013), which help to control fermentation and increase food safety by inhibiting the development of pathogenic bacteria. *Leuc*. spp. are often found in plant matter and food such as fermented meats, fermented vegetables, and fermented dairy products (Holzapfel, Geisen, & Schillinger, 1995; Ortiz et al., 2013).

Ice cream is a processed food that is consumed worldwide by people of all ages. The major structural components of ice cream are fat globules, air, and ice crystals that are dispersed in a frozen solution and concentrated with proteins, salts, and polysaccharides (Muse & Hartel, 2004). Ice cream belongs to the category edible ice cream; however, there are no specifications regarding the concentration of fat, milk protein, or any ingredient (Brasil, 2005). The global ice cream market was valued at $54.8 billion in 2016, and it is expected to reach $78.8 billion by 2025, according to a report by Grand View Research (2018). The demand for lactose‐free products is also expected to contribute to the market growth during the forecast period. The increase in demand for lactose‐free ice creams has been driven by the increase in the incidences of food intolerances and allergies, as well as the preference for lactose‐free diets by a large segment of the population (Grand View Research, 2018).

The development of food products with low fat concentrations has accompanied the new consumer market tendency toward the consumption of foods that facilitate the maintenance of good physical health (da Silva, Siqueira, Binoti, Nunes, & Stephani, 2017). In addition to reduce the fat concentrations in industrial products, the food industry has been concerned about products with reduced lactose concentrations, given that 60%–70% of the world's population is estimated to have lactose intolerance (Heine et al., 2017). Lactose intolerance is associated with a lack of lactase production by the body. The result is symptoms such as swelling, abdominal pain, and diarrhea (Suarez, Savaiano, & Levitt, 1995).

The aim of this study was to develop and to characterize a low‐fat lactose‐free ice cream elaborated by the use of yam‐fermented dough in place of milk.

## 2 MATERIAL AND METHODS

### 2.1 Ice cream elaboration

Approximately 2 kg yam (purchased in the local Market of Lavras) was washed, peeled, and sliced for ice cream preparation. The yam slices were preheated at 60°C for 10 min (Bhandari & Kawabata, 2006). The yam‐based medium was composed of 40% (w/v) preheated yam, 60% (v/v) mineral water, and 1% (w/v) carboxymethylcellulose, according to the previous tests (data not shown). The yam‐based medium was pasteurized at 65°C for 30 min (Bhandari & Kawabata, 2006).

For fermentation, the *Leuc. lactis* strain was selected because of its ability to produce amylase, phytase, reduce pH, and growth in the yam medium (data not shown). The *Leuc. lactis* CCMA0415 strain was grown in a De Man, Rogosa, and Sharpe broth medium (MRS; Merck) at 35°C for 24 hr (10^8^ CFU/ml). After, the cells were inoculated in a yam‐based medium at 10^7^ CFU/ml. The fermentation was performed at 35°C for 6 hr.

The resulting fermented yam dough, as well as the unfermented yam dough, was used for the ice cream preparation. The fermented and unfermented yam dough was added to 0.6% (w/v) stabilizer (Liga neutra; Selecta), 1.11% (w/v) glucose, and 10% (w/v) sucrose. The mixtures were homogenized and then matured for 24 hr. After this period, 0.6% (w/v) emulsifier (Emustab Selecta) was added, and the doughs were beaten in an ice cream maker for 10 min and then frozen at −20°C (Jimenez‐Flores, Klipfel, & Tobias, 1992). Cell viability was evaluated during 90 days of storage by plating in an MRS agar medium (Merck).

### 2.2 Determination of overrun values

The overrun was evaluated according to Jimenez‐Flores et al. (1992). The weight of the ice cream mixture and the weight of the ice cream were obtained and calculated according to Equation 1 below:

(1) $$\text{Overrun}\left(\%\right)=100\times \frac{\text{Volume}\;\text{of}\;\text{ice}\;\text{cream}\;\text{mixture}-\text{Volume}\;\text{of}\;\text{ice}\;\text{cream}}{\text{Volume}\;\text{of}\;\text{ice}\;\text{cream}}$$

### 2.3 Melting test

The ice cream melting was evaluated according to Soukoulis, Chandrinos, and Tzia (2008). The ice cream samples were kept in a freezer at −15°C for approximately 12 hr. Samples of 50 g of ice cream were then transferred to a 2 mm sieve at room temperature (approximately 20°C). The dripped portion was evaluated every 5 min over 105 min. The melting rate was determined as the slope of the graphs of the dripped portion (weight) as function of the time, and expressed in g.min^‐1^.

### 2.4 Physical–chemical composition of the ice cream

The moisture content, protein, lipids, and ash were analyzed according to the AOAC method (AOAC, 2005). The starch content was evaluated by the methodology described by Somogyi–Nelson (Nelson, 1944; Somogyi, 1945).

### 2.5 Mineral content determination

Two grams of ice cream was digested with nitric and perchloric acids (2:1) (v/v). The minerals (Ca, K, Mg, Fe, Zn, P, S, Cu, and Mn) were determined by atomic absorption spectrophotometry and flame photometry, following the method described by Malavolta, Vitti, and Oliveira (1997). The results were based on dry weight.

The molar ratio of phytate to calcium, zinc, and iron was calculated to evaluate bioavailability of ice cream mineral (Phytate/Minerals; Omoruyi, Dilworth, & Asemota, 2007). Phytate was quantified in a previous study (unfermented: 54.92 mg/100 g; fermented: 9.79 mg/100 g; Batista, Ramos, Vilela, Dias, & Schwan, 2019).

### 2.6 Evaluation of total polyphenol content

The total polyphenol content (TPC) of the fermented and unfermented ice creams was evaluated spectrophotometrically according to the Folin–Ciocalteu method (Singleton & Rossi, 1965). Ice cream samples of 0.5 ml were homogenized with 2.5 ml (10%) Folin–Ciocalteu reagent and 2.0 ml (4% w/v) sodium carbonate (Na_2_CO_3_) and stored in the dark for 2 hr. The absorbance of the samples was evaluated at 750 ηm. The TPC content was obtained through a standard curve of gallic acid. The results were based on wet weight and expressed as mg of gallic acid/100 g of ice cream sample. The analyses were performed in triplicate.

### 2.7 Determination of free radical activity by the 1,1‐diphenyl‐2‐picrylhydrazyl and 2,2′‐azino‐bis (3 ethylbenzo‐thiazoline‐6‐sulfonic acid) methods

The radical scavenging activity (RSA) of the fermented and unfermented ice creams was evaluated using the radicals 1,1‐diphenyl‐2‐picrylhydrazyl (DPPH) and 2,2′‐azino‐bis (3 ethylbenzo‐thiazoline‐6‐sulfonic acid) (ABTS) (Brand‐Williams, Cuvelier, & Berset, 1995; Re et al., 1999). The storage solutions of ABTS (7 mM) and potassium persulfate (140 mM) were homogenized and kept in the dark at room temperature for 16 hr. The ABTS solution was diluted into ethanol until an absorbance of 0.70 (±0.05) at 734 ηm was achieved. Aliquots of 30 μl ice cream were added to 3.0 ml ABTS solution. After 6 min, the absorbances were registered. The control was evaluated using 30 μl ethanol as a replacement for the ice cream.

The DPPH was evaluated by the homogenization of 0.1 ml ice cream with 3.9 ml DPPH solution (0.06 mM), followed by incubation in the dark for 2 hr. Absorbance was achieved at 515 ηm. The control was evaluated using 0.1 ml methanol as a replacement for the ice cream.

The RSA was calculated for all of the samples according to Equation 2:

(2) $$\%\text{RSA}=100\times \frac{Ab-As}{\mathrm{Ab}}$$

where *Ab* is the control absorbance and *As* is the sample absorbance.

### 2.8 Fluorescence microscopy

The fluorescence analyses were made with the Zeiss Axio‐Observer Z1 LSM 780 laser confocal microscope and Zen 2010 software (Carl Zeiss Microscopy MBH) at the Electron Microscopy Laboratory at the Federal University of Lavras. Syto9 fluorochrome (Thermo Fischer^®^) was used to label live bacterial cells (ChS1 detector), and a propidium iodide (PI) solution (Sigma^®^) was used to mark the dead bacteria (Ch2 detector; Stiefel, Schmidt‐Emrich, Maniura‐Weber, & Ren, 2015; Zotta, Guidone, Tremonte, Parente, & Ricciardi, 2012). The nonfluorescent images were observed with the TPMT ChD detector. In brief, 5 µl of each sample was deposited above 0.25 cm^2^ water‐agar blocks and kept still for 5 min. On the same surface, 5 µl Syto9 (20 µM) was applied, and the mixture was incubated in the dark for 40 min. Next, 5 µl PI (1 µg/ml) was incubated for 5 min on dark. The images were edited with FIJI ImageJ and Corel Draw software.

### 2.9 Rheological behavior

The rheological test was performed at 5°C with the rheometer HAAKE RheoStress 6000 (Thermo Scientific) equipped with a HAAKE A10 (Thermo Scientific) thermostatic water bath and a universal temperature control system HAAKE UTM controller (Thermo Scientific). A set of concentric cylindrical geometry sensors (CCB25 DIN cup and CC25 DIN Ti rotor) with a gap of 5.3 mm was used.

Each sample was subjected to a continuous deformation rate ramp of 0–300 s^−1^ for 2 min for the upward curve and 2 min for the downward curve to break the thixotropy and eliminate the influence of time on the flow behavior of the treatments. After this procedure, the flow curve was generated for the rheological characterization of each sample by the application of a flow curve varying the deformation rate from 0 to 300 s^−1^ for 3 min. Newton's law (Equation 3), the power law (Equation 4), and the Herschel–Buclkey model (Equation 5) were fitted to the experimental data of the flow curves.

(3) σ=μγ

(4) $$\sigma =K\gamma^{n}$$

(5) $$\sigma =\sigma_{0}+K\gamma^{n}$$

where σ is the shear stress (Pa), μ is the Newtonian viscosity (Pa·s), γ is the deformation rate (s^−1^), *K* is the consistency index (Pa.sn), *n* is the flow behavior index (dimensionless), and σ_o_ is the initial tension (Pa).

### 2.10 Statistical analysis

The results were evaluated with analysis of variance (ANOVA) and Tukey's range test (Tukey, *p* < 0.05) using the Statistical Analysis System (SAS) software (University Edition, 2016). The rheological parameter models were fitted to the experimental data of the flow curves using the SAS software, and the graphs were plotted using SigmaPlot 11.0 software (Systat Software Inc., 2008).

## 3 RESULTS AND DISCUSSION

The ice creams were elaborated with fermented and unfermented yam. The moisture, ash, protein, lipid, and starch contents were evaluated (see Table 1). The ice creams consisted of 86%–87% moisture, 0.2%–0.22% ash, 3.9%–4.6% protein, 0.16%–0.17% lipids, and 22%–26% starch. The protein and starch content in the fermented samples was significant decreased (*p < *0.05). The reason might have been the protease and amylase activity of the *Leuc. lactis* CCMA0415 starter culture. This strain was previously characterized as an amylase producer (data not shown). It is likely that peptides were used as a source of nitrogen by the microorganism. Amylase hydrolyzes the starch into smaller polymers, thereby being essential enzyme for microorganisms which uses starch as a carbon and energy source. The *Leuconostoc* genera have been described as protease producers (Sonar & Halami, 2014).

**Table 1:** Physical–chemical composition of unfermented and fermented yam‐based ice creams

Parameters	Concentration (%)	
	Unfermented	Fermented
Moisture	87.25 ± 0.09^a^	86.46 ± 0.21^a^
Ash	0.22 ± 0.0^a^	0.2 ± 0.01^a^
Protein	4.68 ± 0.01^b^	3.99 ± 0.09^a^
Lipid	0.17 ± 0.0^a^	0.16 ± 0.0^a^
Starch	26.82 ± 0.0^b^	22.35 ± 0.0^a^

Note

Mean value ± standard deviation. Means followed by the same letter in the lines did not differ with Tukey test (*p* < 0.05).

The concentrations of the same minerals in fermented and unfermented yam ice creams were also evaluated (see Table 2). The concentrations of the minerals K, S, Cu, Mn, Zn, and Fe (13.0, 0.9, 4.4, 0.56, 11.19, and 21.45, respectively) were significantly higher (*p < *0.05) in the fermented ice cream than in the unfermented product (11.4, 0.6, 4.2, 0.15, 11.07, and 18.11, respectively). The increased concentrations of minerals in the cereals, roots, and tubers fermented by the LAB were probably the result of fermentation process (Digbeu, Due, & Dabonne, 2013). A bioavailability of Fe, Zn, and Ca was evaluated in unfermented (2.57, 4.91, and 0.17, respectively) and fermented (0.39, 0.87, and 0.02, respectively) ice creams. The desirable levels for mineral absorption are phytate:iron (<1), phytate:zinc (<18), and phytate:calcium (<0.17; Gibson, Bailey, Gibbs, & Ferguson, 2010). The improvement in the bioavailability of these minerals was probably due to the phytase activity. This enzyme hydrolyzes phytic acid, thus preventing the formation of stable complexes with the minerals (Ghosh et al., 2015; Lopez, Leenhardt, Coudray, & Remesy, 2002). The use of LAB strains in vegetable‐based fermentations has been shown to be efficient for increasing mineral bioavailability (Anastasio et al., 2010).

**Table 2:** Mineral concentration of unfermented and fermented yam‐based ice creams

Minerals	Unfermented	Fermented
P (g/L)	0.7 ± 0.04^a^	0.8 ± 0.02^a^
K (g/L)	11.4 ± 0.05^a^	13.0 ± 0.28^b^
Ca (g/L)	0.2 ± 0.04^a^	0.3 ± 0.02^a^
Mg (g/L)	0.5 ± 0.05^a^	0.5 ± 0.01^a^
S (g/L)	0.6 ± 0.02^a^	0.9 ± 0.02^b^
Cu (mg/L)	4.2 ± 0.04^a^	4.4 ± 0.04^b^
Mn (mg/L)	0.15 ± 0.02^a^	0.56 ± 0.02^b^
Zn (mg/L)	11.07 ± 0.02^a^	11.19 ± 0.02^b^
Fe (mg/L)	18.11 ± 0.05^a^	21.45 ± 0.05^b^

Note

Mean value ± standard deviation. Means followed by the same letter in the lines did not differ with the Tukey test (*p* < 0.05).

**Figure 1:**
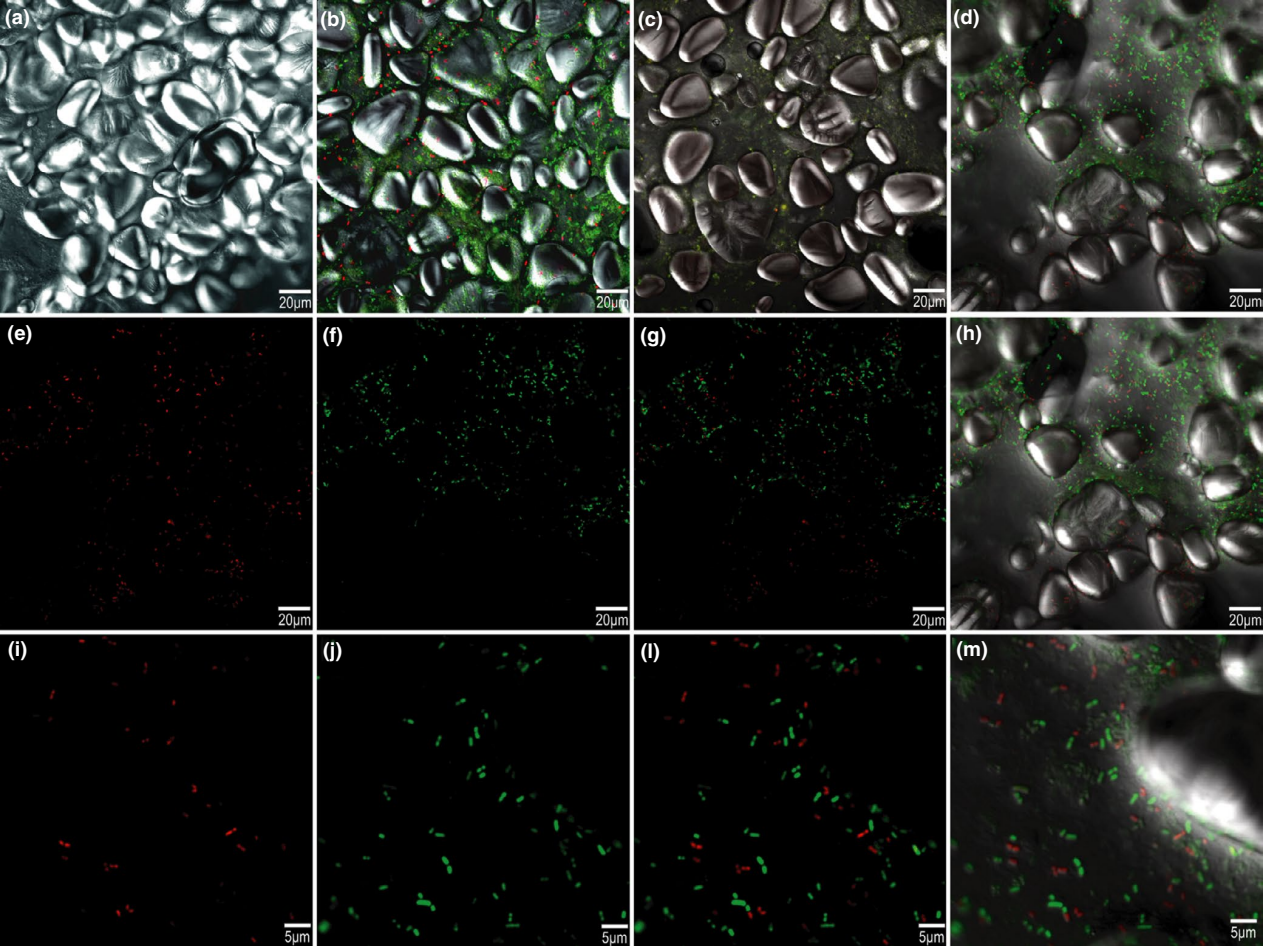
Fluorescence microscopy of the ice cream mixture and the ice cream (a–l). (a) unfermented ice cream mixture; (b) fermented ice cream mixture; (c) unfermented ice cream; (d, h, and l) fermented ice cream mixture; (e and i) dead cells (red fluorescence); (f and j) living cells (green fluorescence); (g and k) dead and living cells; (b, d, h, and l) dead and living cells. (a–h) 38× magnification; (i–l) 126× magnification

Cell viability can be achieved by either the plating method or fluorescence microscopy. The fluorescence microscopy analysis discriminates between live and dead cells (Stiefel et al., 2015; Zotta et al., 2012), which can be observed by the emission of red fluorescence. In contrast, living cells are characterized by green fluorescence (see Figure 1).

The intensity of the fluorescence was evaluated in the fermented ice cream. The living cells showed higher fluorescence intensity (0.696) than the dead cells (0.031) because of the high viable bacterial population present in the ice cream. The *Leuc. lactis* population was evaluated by the plating method. Despite the reduction of approximately 1 log CFU/mL, the population remained high; thus, the results were in agreement with those obtained through the microscopy analysis.

The observed cell death could likely have resulted from the freezing process, which can damage the cell membrane of the bacteria and lead to injuries that compromise cell function and metabolic activity (Tripathi & Giri, 2014; Vogels et al., 1992).

In addition to cell viability, higher starch concentration in ice cream unfermented mixture (Figure 1a) regarding ice cream fermented mixture (Figure 1b) was observed. Moreover, the process of incorporating air into the ice cream mixture showed that there was homogeneous dispersion of the starch and the *Leuc. lactis* CCMA0415 cells throughout the ice cream matrix (see Figure 1c,d).

The ice cream was analyzed during 90 days of storage, and the *Leuc. lactis* CCMA0415 population was observed and evaluated by the plating method. The population remained constant (~ 7 log CFU/mL) as did the titratable acidity (~ 0.2%) and the pH (~ 4.0) (see Table 3). The high viability of the *Leuc. lactis* CCMA0415 cells could likely be attributed to their peptidoglycan composition, as suggested by Polo et al. (2017), who reported the presence of meso‐diaminopimelic acid in the cell wall of LAB with a freezing tolerance of −196°C.

**Table 3:** Lactic acid concentration, pH values, and *Leuconostoc lactis* CCMA0415 population during 90 days of storage of unfermented and fermented yam‐based ice creams

Time (days)	Lactic acid concentration (%)		pH		*Leuc. lactis* (CFU/ml)
	Unfermented	Fermented	Unfermented	Fermented	Fermented
0	ND	ND	6.01 ± 0.00	6.01 ± 0.05	7.70 ± 0.02
7	ND	0.23 ± 0.0	6.05 ± 0.02	3.93 ± 0.04	7.30 ± 0.65
14	ND	0.21 ± 0.0	6.00 ± 0.01	4.00 ± 0.00	7.34 ± 0.25
21	ND	0.22 ± 0.0	6.08 ± 0.02	4.13 ± 0.01	7.04 ± 0.41
30	ND	0.21 ± 0.0	5.975 ± 0.00	4.06 ± 0.00	6.80 ± 0.47
60	ND	0.21 ± 0.0	6.07 ± 0.00	4.13 ± 0.01	7.33 ± 0.50
90	ND	0.23 ± 0.0	6.10 ± 0.00	4.05 ± 0.00	6.51 ± 0.06

Note

Mean ± standard deviation.

Abbreviation: ND: not detected.

The TPC and antioxidant activity were evaluated in the yam‐based ice creams (Table 4). The TPC was 51–54 mg/g. No significant difference (*p > *0.05) was observed between the ice creams. However, the antioxidant activity with the DPPH and ABTS methods was significantly different (*p < *0.05), with lower values being observed for the fermented samples than for the unfermented samples. These results could indicate the possible interaction of plant phenolics with microbial proteins that can form insoluble complexes and reduce ice cream antioxidant activity (Liu, Chen, Shao, Wang, & Zhan, 2017).

**Table 4:** TPC and antioxidant activity in unfermented and fermented ice creams as evaluated by DPPH and ABTS methods

	TPC (mg/g of ice cream)	DPPH (%)	ABTS (%)
Unfermented	0.54 ± 0.01^a^	18.36 ± 0.09^a^	44.31 ± 0.3^a^
Fermented	0.51 ± 0.03^a^	10.64 ± 0.00^b^	26.31 ± 0.4^b^

Note

Mean value ± standard deviation. Means followed by the same letter in the lines did not differ with the Tukey test (*p* < 0.05).

The free radical reduction capacity with the DPPH method was 18% and 10% for the fermented and unfermented ice creams, respectively. With the ABTS method, it was 44% and 26% for the fermented and unfermented ice creams, respectively. In an evaluation of the free radical removal capacity of yam mucilage, Zhang, Wang, Liu, and Li (2016) obtained values of 25%–80%, thus demonstrating that yam mucilage contributes to antioxidant activity of yams.

**Figure 2:**
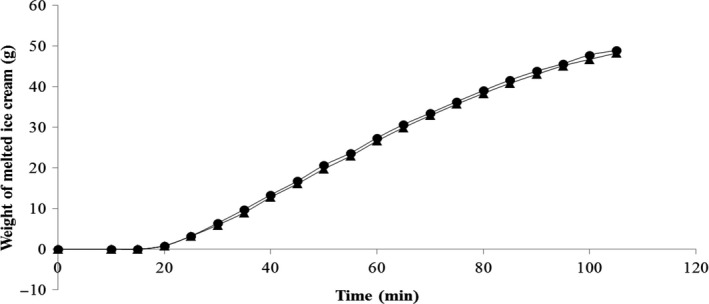
Melting curve of unfermented (●) and fermented (▲) yam‐based ice creams

Figure 2 shows the melting behavior of the yam‐based ice creams and the melting rate determined by linear regression (*r^2^* = 0.98). The melting rate, approximately 0.5 g/min, was not significantly different for the fermented and unfermented samples. The low melting rate obtained was probably the result of the low concentrations of fats used for the elaboration of the ice creams because the net of fat globules formed during freezing influences the melting rate (Muse & Hartel, 2004). The reduced melting rate was also observed by Sharma, Singh, and Yadav (2017) in ice cream with a low concentration of fat elaborated with octenyl succinylated pearl millet starch. Melting can also be influenced by overrun and the structure of the ice crystals (Muse & Hartel, 2004). Fat reduction is yet another factor in the overrun rate. Moriano and Alamprese (Moriano & Alamprese, 2017) observed that fat concentrations and air incorporation are inversely proportional. This fact could justify the high overrun rate: 233% and 283% for the unfermented and fermented ice creams, respectively.

Another factor in the overrun rate was likely the high protein concentration (approximately 4% in the yam‐based ice creams). Chang and Hartel (2002) stated that, in addition to fat, the proteins and hydrocolloids aid in air incorporation and the thermodynamic control of air bubbles. Although the overrun rates for the yam‐based ice creams were high, they were in accordance with those proposed by Flores and Goff (1999), who suggested that ice cream should have a 70% or higher overrun rate. This value is enough to avoid collisions among the ice crystals and to keep the serum phase dispersed around each crystal. Furthermore, the results suggest that the low melting rate and the high overrun value could also be related to the reduced heat transfer rate caused by the large volume of air (Sofjan & Hartel, 2004).

**Figure 3:**
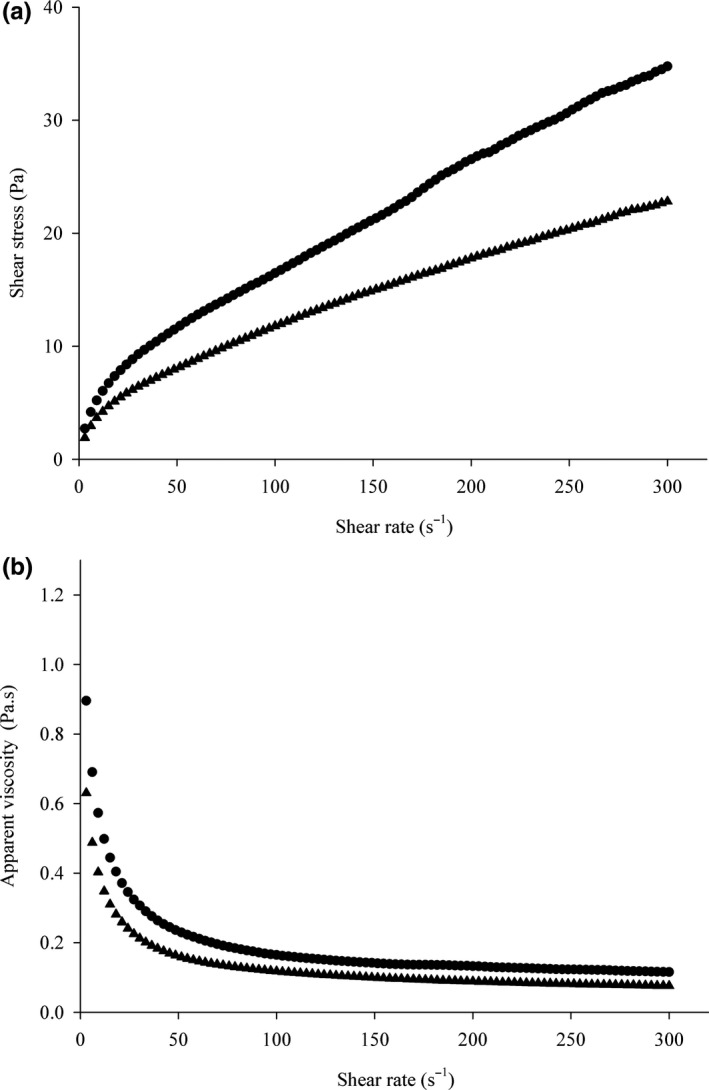
Rheological behavior: (a) relationship between the shear stress (Pa) and the strain rate (s^−1^); and (b) the relationship between the apparent viscosity (Pa s) and the strain rate (s^−1^) of unfermented (●) and fermented (▲) yam‐based ice creams

The rheological behavior of the fermented and unfermented yam‐based ice creams was evaluated (see Figure 3). Of the models used (see Table 5), the power law showed the most appropriate adjustments to the experimental data, with high coefficient of determination (*r* ^2^ = 0.99) and low mean squared error values; however, no significant difference was observed between the fermented and unfermented ice creams.

**Table 5:** Rheological parameters of emulsions

Samples	Power law			
	*K*	*n*	RMSE	*R* ^2^
Unfermented	1.3921 (± 0.1705)^a^	0.6032 (± 0.0236)^a^	0.7775	0.9912
Fermented	0.8533 (± 0.3167)^a^	0.5726 (± 0.0245)^a^	0.8652	0.9801

Note

Mean ± standard deviation; *n* = 4. Means followed by the same letter in the lines did not differ with the Turkey test (*p* < 0.05). *K* represents the consistency index (Pa·s*^n^*), *n* represents the flow behavior index (dimensionless), RMSE is the mean squared error, and *R* ^2^ is the coefficient of determination.

The fermented and unfermented ice creams were characterized as non‐Newtonian fluids, and they exhibited pseudoplastic behavior (*n* < 1; see Figure 3). The *n* values were 0.58–0.60; no significant differences were observed (*p > *0.05).

In general, the ice creams with low‐fat content had *n* values of 0.43–0.66 (Aime, Arntfield, Malcolmson, & Ryland, 2001). The fermented ice cream showed higher shear stress (≅35 Pa) than the unfermented ice cream (≅21 Pa; see Figure 3). The shear stress might have been affected by food composition, homogenization pressure, air incorporation, and ice crystal aggregation (Kus, Altan, & Kaya, 2005; Sofjan & Hartel, 2004). Thus, this variation could be the result of the significant differences found in the starch and protein content of the fermented and unfermented ice cream samples.

The viscosity of the ice cream samples decreased with the rate of deformation (see Figure 3b), and the lowest viscosity was observed for the fermented yam‐based ice cream. It is known that the carbohydrate concentration affects the viscosity of a solution because of its ability to bind to the water molecules, thereby increasing the viscosity (Aykan, Sezgin, & Guzel‐Seydim, 2008; Schmidt, Lundy, Reynolds, & Yee, 1993). The fermented sample had lower concentrations of starch, and this might have resulted in the lower viscosity.

## 4 CONCLUSION

Ice creams containing low fat concentrations were successfully elaborated from fermented and unfermented yam dough. According to the obtained data, the fermentation of yam seems to improve mineral availability and decrease starch and protein content of ice cream, and this could lead to higher digestibility. *Leuc. lactis* CCMA 0415 was successfully used as a starter culture for the yam fermentation. The fermented and unfermented ice creams were characterized as non‐Newtonian with pseudoplastic behavior. Therefore, yam was characterized as a suitable raw material substitute for milk during the elaboration of edible ice cream.

## CONFLICT OF INTEREST

The authors declare no conflict of interest.

## ETHICAL STATEMENTS

This study does not involve any human or animal testing.
